# Analysis of phase II methodologies for single-arm clinical trials with multiple endpoints in rare cancers: An example in Ewing’s sarcoma

**DOI:** 10.1177/0962280216662070

**Published:** 2016-09-01

**Authors:** P Dutton, SB Love, L Billingham, AB Hassan

**Affiliations:** 1 Centre for Statistics in Medicine (CSM), University of Oxford, Botnar Research Centre, Oxford, UK; 2 Nuffield Department of Orthopaedics, Rheumatology and Musculoskeletal Sciences (NDORMS), University of Oxford, Nuffield Orthopaedic Centre, Oxford, UK; 3 Cancer Research Clinical Trials Unit (Cancer Sciences), School of Cancer Sciences, College of Medical and Dental Sciences, University of Birmingham, Birmingham, UK; 4 Oxford Molecular Pathology Institute, Sir William Dunn School (OMPI, SWDS), University of Oxford, Oxford, UK

**Keywords:** Bayesian clinical trial, phase II; multi-stage design, early stopping, multiple endpoints

## Abstract

Trials run in either rare diseases, such as rare cancers, or rare sub-populations of common diseases are challenging in terms of identifying, recruiting and treating sufficient patients in a sensible period. Treatments for rare diseases are often designed for other disease areas and then later proposed as possible treatments for the rare disease after initial phase I testing is complete. To ensure the trial is in the best interests of the patient participants, frequent interim analyses are needed to force the trial to stop promptly if the treatment is futile or toxic. These non-definitive phase II trials should also be stopped for efficacy to accelerate research progress if the treatment proves to be particularly promising. In this paper, we review frequentist and Bayesian methods that have been adapted to incorporate two binary endpoints and frequent interim analyses. The Eurosarc Trial of Linsitinib in advanced Ewing Sarcoma (LINES) is used as a motivating example and provides a suitable platform to compare these approaches. The Bayesian approach provides greater design flexibility, but does not provide additional value over the frequentist approaches in a single trial setting when the prior is non-informative. However, Bayesian designs are able to borrow from any previous experience, using prior information to improve efficiency.

## 1 Introduction

One of the biggest challenges for clinical trials for rare diseases and rare subsets of common diseases is patient recruitment.^1^ When the patient population is very small, it is often not feasible to run a traditional fully powered phase II trial in a reasonable time frame and it is impossible to repeat this in a phase III confirmatory setting. Likewise, as the population of candidates is so small, running a trial with a small chance of success (based on the trial data so far) hinders research by consuming patients who would provide more useful information if they were recruited to more promising trials that aimed to answer more decisive questions.

Research is increasingly focusing on personalised medicine, which is the customisation of treatment often using genetics as a prognosis tool for treatments. For example, in cancer, the activity and clinical benefit of several agents mechanistically depend on specific mutations in genes that drive the cancer.^2^ Even in a given histological subtype of a more common disease, some treatments may only work in a sub-population due to interactions between a patient’s genes and treatment. It may therefore not be economical to treat all patients with a drug if there is no benefit for those without a specific biomarker that is correlated with response. The general problems that research into rare diseases faces are also relevant for research into the treatment of small sub-populations of more common diseases.

Drugs are not often developed for rare diseases as it is historically more profitable to target common diseases. Academic research questions often fill this gap, focusing on rare diseases and subtypes identified through mechanistic studies, hypothesis testing and translational research. This research can and should inform the clinical trial question, and may also enhance the potential impact of success at the clinical trial stage. Separation of mechanistic research and clinical trials is deemed a weakness in the development strategies of larger pharmaceutical companies.^3^ As early phase dose finding studies are often performed with patients in a more common (or general) disease setting, interactions between the treatment and rare disease may not be well understood. It is therefore important to have careful management of toxicity built into the design of a trial which relies on such phase I trials but in an untested rare cancer setting. The most robust approach is to formally consider the toxic event rate as a co-primary endpoint alongside patient response and to allow for stopping at interim analyses if the observed toxicity rate is too high. Data safety monitoring committees (DSMC) are entrusted with the role of ensuring trials only continue if they are in the interest of patients in the clinical trial setting. However, DSMCs tend to have limited experience in rare disease settings, so need additional guidance on acceptable toxicity rates.

We review a number of frequentist and Bayesian methods that have been adapted to incorporate two binary endpoints and frequent interim analyses. The LINES trial is used as a motivating example and provides a suitable platform to compare these approaches.

## 2 Methods

The question we set out to address concerns optimising the trial design for a phase II trial in this setting of rare selected patient groups with co-primary endpoints: efficacy and toxicity. We consider the following criteria to be critical to this question from the clinical trial perspective:

- Small maximum sample size to ensure that answers can be obtained in a reasonable period
- Formal ability to stop the trial early if the treatment is ineffective
- Formal ability to stop the trial early if the treatment is toxic
- Formal ability to stop the trial early if the treatment is effective
- Small expected sample size if the treatment is ineffective, toxic or both
- Controlled type I error for both futility and toxicity
- Reasonable power to observe efficacy, provided the toxicity is acceptable

For the purpose of this paper, a patient will either respond or not respond to treatment and a patient will also either experience or not experience a toxic event, both of which are binary endpoints. Let $\theta^{R}$ be the probability of a patient having a response. The hypotheses for a response-only trial are

$$\begin{array}{c} H_{0}:\theta^{R}<\theta_{0}^{R}\\ H_{1}:\theta^{R}>\theta_{0}^{R} \end{array}$$

where $\theta_{0}^{R}$ is some uninteresting probability of response. The alternative hypothesis is traditionally given a clinically interesting probability $\theta_{1}^{R}$ ($\theta_{1}^{R}>\theta_{0}^{R}$), whereby if the probability of response was at least this large the investigators would certainly be interested in investigating the treatment further. Under the frequentist approach, we wish the type I error to be less than α (the probability that further research is recommended when the true response rate is at most $\theta_{0}^{R}$) and the type II error to be less than β (the probability that further research is rejected when the true response rate is at least $\theta_{1}^{R}$). Thus the power must be at least 1-β.

If we include toxicity in the trial design, letting $\theta^{T}$ be the probability of a patient experiencing a toxic event, the hypotheses become

$$\begin{array}{c} H_{0}:\theta^{R}<\theta_{0}^{R}\text{or}\theta^{T}>\theta_{0}^{T}\\ H_{1}:\theta^{R}>\theta_{0}^{R}\text{ and }\theta^{T}<\theta_{0}^{T} \end{array}$$

Thus both high efficacy and low toxicity are needed to recommend further research.

Frequentist methods are based on simulations that assume the null and alternative hypotheses are true. The type I and type II errors encapsulate the likelihood of a given design giving the wrong result, if the null or alternative hypotheses are true. In addition to this the expected sample size given a scenario is also a frequentist property. The type I error can also be considered with respect to just one endpoint allowing more direct comparisons between single endpoint designs and multi-endpoint designs.

Bayesian methods are based on current information, which is captured in a posterior distribution that incorporates any prior knowledge with currently observed trial data. We denote the posterior distribution of response and toxicity as $\Theta^{R}$ and $\Theta^{T}$. Within the trial, commonly used Bayesian properties for this problem are the following posterior probabilities: $P(\Theta^{T}\leq \theta_{0}^{T}|\text{data},\text{prior})$, $P(\Theta^{T}\geq \theta_{1}^{T}|\text{data},\text{prior})$, $P(\Theta^{R}\geq \theta_{0}^{R}|\text{data},\text{prior})$ and$P(\Theta^{R}\leq \theta_{1}^{R}|\text{data},\text{prior})$. These correspond to no toxicity, toxicity, efficacy and futility.

The interpretation of these properties is as follows:

- *Type I error*: The probability of rejecting the null hypothesis (*H*_0_) when it is true, or the largest probability of rejecting *H*_0_ under the three null hypothesis scenarios, $(\theta_{0}^{R},$ $\theta_{0}^{T})$, ($\theta_{0}^{R},$ $\theta_{1}^{T}$) and$(\theta_{1}^{R},$ $\theta_{0}^{T})$.
- *Type II error*: The probability of rejecting the alternative hypothesis (*H*_1_) when it is true.
- *Expected sample size under a specific scenario*: The expected sample size by simulation, given the probabilities$\left(\theta^{R},\theta^{T}\right)$.
- *Bayesian posterior probabilistic properties*: The probability that the true value is in a specific range, based on all known information (current data and prior information incorporated into the posterior distribution). For each endpoint there are two values: the posterior probability of being superior to the null hypothesis (e.g. $\theta_{0}^{R})$, and the posterior probability of being inferior to the level assigned to the alternative hypothesis (e.g. $\theta_{1}^{R}$).

Having two endpoints may increase the sample size required to achieve the same type I and II errors. To control for type I and type II errors in a two-endpoint setting, the data must be considered under four scenarios: type I error for the probability pairs$(\theta_{0}^{R},$ $\theta_{0}^{T})$, ($\theta_{0}^{R},$ $\theta_{1}^{T}$) and $(\theta_{1}^{R},$ $\theta_{0}^{T})$, and type II error for the probability pair$(\theta_{1}^{R},$ $\theta_{1}^{T})$. It is easy to see that the type I error for the first pair is always smaller than the type I error for the second and third pairs. It may also be useful to know the type I and type II errors under the assumption that there is no toxicity. This is directly synonymous with the single-endpoint trial, and is a good way of examining the effect of the second endpoint on the design characteristics.

There are a number of both frequentist and Bayesian approaches to the problem of a single-arm two-endpoint phase II trial in this setting. We evaluate two frequentist and three Bayesian approaches which allow two endpoint designs. We discuss the frequentist and Bayesian properties of each method, so that they can be directly compared.

These methods are compared on the LINES trial, a phase II trial in Ewing’s sarcoma patients with relapsed or metastatic disease.

## 3 Trial designs

The response and toxicity endpoints are binary. The data will come from a $\text{binomial}(n,\theta^{R})$ distribution for the response endpoint and a $\text{binomial}(n,\theta^{T})$ distribution for the toxicity endpoint. The endpoints will be considered independent from each other. The response endpoint can be discussed in terms of futility, when the response is insufficient, and in terms of efficacy, when the response is sufficient. The treatment must be seen to be both efficacious and non-toxic in order to recommend further research at each analysis.

## 4 Frequentist approaches

### 4.1 Single-stage

The single-stage, single-endpoint frequentist trial is the oldest tried-and-tested trial design. There is no formal interim analysis. Adjustments to the sample size can be made to formalise the intention to test two endpoints at the same time.

### 4.2 Bryant and Day’s adaptation of Simon’s two-stage design

Simon’s two-stage design is one of the most influential frequentist designs: it is one of the first trial designs to actively look at a binary endpoint (response) for a trial at an interim analysis. Simon computed exact sample sizes for a two-stage design that could stop for futility at interim. Fleming^4^ had previously computed sample sizes for this design using Gaussian asymptotics. Simon’s method reduces the expected sample size when the treatment of interest is insufficiently efficacious. Simon proposed two methods of optimisation, optimal design, which minimises the expected sample size under the null hypothesis (*H*_0_), and minmax, which minimises the maximum sample size. Simon noted that it is preferable to cease recruitment once the required number of patients for the interim analysis have been recruited. The interim analysis thus has the required number of patients and has the properties of the designed trial. The properties will differ if recruitment is not stopped.

Simon’s two-stage design^5^ set the groundwork for a number of papers extending his design. Bryant and Day^6^ proposed including toxicity as a co-primary endpoint, which incorporated toxicity into Simon’s two-stage design. Their approach allows stopping at interim analysis for toxicity or futility. They showed that error rates were controlled even when the assumption of independence between the two endpoints was relaxed.

Further work on multiple endpoints was done by Conway and Petroni.^7,8^ They allowed for a trade-off between response and toxicity.

### 4.3 Lan-Demets alpha spending approach

The Lan-Demets alpha spending approach^9^ is a generalised approach to multiple interim analysis for frequentist designs. It allows more of the type I and/or type II error to be spent at later interim analyses using an alpha spending function, such as the O’Brien–Fleming^10^ alpha spending function. This adjustment inflates the type I error slightly, but much less than testing repeatedly at the full type I error level alpha. As no work has been published on extending this methodology to multiple endpoints, it has not been included.

## 5 Bayesian methodology

The data are modelled using independent binomials. The beta distribution makes an ideal prior as it is conjugate. The posterior is thus also a beta distribution

$$\begin{array}{c} \Theta_{\text{prior}}^{R}∼\text{Beta}(\alpha^{R},\beta^{R})\\ X^{R}∼\text{Binomial}(n,R)\\ \Theta_{\text{posterior}}^{R}∼\text{Beta}(\alpha^{R}+x^{R},\beta^{R}+n-x^{R})\\ \Theta_{\text{prior}}^{T}∼\text{Beta}(\alpha^{T},\beta^{T})\\ X^{T}∼\text{Binomial}(n,\theta^{T})\\ \Theta_{\text{posterior}}^{T}∼\text{Beta}(\alpha^{T}+x^{T},\beta^{T}+n-x^{T}) \end{array}$$

Prior knowledge can be incorporated into the model. This information is often collected via careful prior elicitation meetings between relevant experts. Data from previous studies may be included if relevant, and may be weighted depending on their relevance and quality.^11^ Non-informative priors can be used if no prior information is available. Sensitivity analyses should be performed after concluding a trial that uses informative prior information, to check that the conclusions are robust and that any significant results are not solely created by the prior information.

There are three possible outcomes at each interim analysis: stop for futility and/or toxicity, stop for efficacy and no toxicity or continue to recruit patients. The design must be finalised before recruiting any patients, and is used as a guide during the trial. Although the Bayesian paradigm does not require interim analyses to occur at an exact number of patients, the frequentist properties of the design are affected by the number of patients at each interim analysis.

An alternative modelling approach considers using a Dirichlet prior with multinomial data (two-by-two outcomes for response and toxicity).^12^ This approach allows the dependence between response and toxicity to be added, which is particularly important when the two endpoints are suspected to be dependent as it allows dependent priors to be used. The adaptation is not required when independent priors are chosen.

Whitehead et al.^13^ proposed a Bayesian approach to compute sample size that can be considered a direct translation of the frequentist approach to the Bayesian setting. They considered the posterior probabilities $P(\Theta^{R}\geq \theta_{0}^{R}|\text{data},\text{prior})$ for efficacy and $\left(\Theta^{R}\leq \theta_{1}^{R}|\text{data},\text{prior}\right)$ for futility. They proposed that the evidence be considered convincing if the probabilities were sufficiently large. As a trial should be conclusive when stopped, the sample size *n* is computed such that there exists $x_{C}^{R}$ which satisfies

$$\begin{array}{c} P(\Theta^{R}\geq \theta_{0}^{R}|n,X^{R}=x^{R},\text{prior})>\zeta^{R}\\ P{(\Theta }^{R}\leq \theta_{1}^{R}|n,X^{R}=x^{R}-1,\text{prior})>\eta^{R} \end{array}$$

Here $\zeta^{R}$ and $\eta^{R}$ are the smallest acceptable posterior probabilities to recommend further research or recommend no further research. $x_{C}^{R}$ is then the smallest number of responses required to declare the treatment is effective given n patients are evaluable.

This approach can be extended to multiple endpoints provided there is to be no trade-off between the endpoints at the final analysis.

## 6 Bayesian approaches

### 6.1 Bayesian probabilistic properties approach

This approach defines a series of stopping rules based directly on the Bayesian posterior probabilistic properties ($P(\Theta^{T}\leq \theta_{0}^{T}|n,x^{T},\text{prior})$, $P{(\Theta }^{T}\geq \theta_{1}^{T}|n,x^{T},\text{prior})$, $P(\Theta^{R}\geq \theta_{0}^{R}|n,x^{R},\text{prior})$ and $P(\Theta^{R}\leq \theta_{1}^{R}|n,x^{R},\text{prior})$ both at interim and at the end of the trial. Posterior probabilities are easy to interpret in the context of the original problem. Thresholds are set for each posterior probability. The trial stops when the probability exceeds one of the thresholds. If the trial continues to the final analysis, then further research is only recommended if the threshold is exceeded. A trial can use one or more of the following decision rules to generate a suitable design:

- If $P(\Theta^{T}>\theta_{1}^{T}|n,x^{T},\text{prior})>\eta^{T}$ stop for toxicity
- If $P(\Theta^{R}<\theta_{1}^{R}|n,x^{R},\text{prior})>\eta^{R}$ stop for futility
- If $P(\Theta^{\text{R}}>\theta_{0}^{R}|n,x^{R},\text{prior})>\zeta^{R}$ and if $P(\Theta^{T}<\theta_{1}^{T}|n,x^{T},\text{prior})>\zeta^{T}$ stop for efficacy and no toxicity

These decision rules can be used at both interim and final analysis. The posterior probability thresholds $\eta^{T}$, $\eta^{R}$, $\zeta^{R}$ and $\zeta^{T}$ need not be constant across each analysis and may vary with the current number of patients recruited.

### 6.2 Bayesian posterior predictive probability approach

Herson^14^ first proposed the use of Bayesian predictive probability to make decisions about early termination of clinical trials. The rules for recommending further research at the conclusion of the trial are computed in the same way as for the probabilistic approach above. At an interim analysis with *n_i_* patients, the current posterior distribution is known and hence the posterior predictive distribution can be obtained. The probability of a successful trial is then the probability that the posterior predictive distribution for $n-n_{i}$ patients will achieve at least the threshold for recommending further research at the conclusion of the trial, when *n_i_* patients have data and *n* is the maximum number of patients to recruit. The trial will stop for futility if the probability of a successful trial is sufficiently low and for efficacy if it is sufficiently high. If it is neither high nor low, then the evidence is inconclusive and the trial continues to recruit patients.

This approach is easily extended to multiple endpoints. Each endpoint can have a decision rule. All of the endpoints must be sufficiently likely to attain at least the critical value for the trial to stop early for success. It is also possible, if desired, to stop early for futility if the probability of hitting just one of the endpoints is particularly low. This provides additional degrees of freedom to the design and is useful if one endpoint (such as toxicity) is of greater concern to investigators during early stages of the trial.

The thresholds can then be adapted to generate a trial with the desired properties. A trial can use one or more of the following decision rules to generate a suitable design:

- If $P{(\text{X}}_{\text{predictive}}^{\text{T}}>x_{c}^{T}-x_{i}^{T}|n_{i},x_{\text{i}}^{\text{T}},\text{prior})>\eta^{T}$ at interim, then stop for toxicity
- If $P{(\text{X}}_{\text{predictive}}^{\text{R}}<x_{c}^{R}-x_{i}^{R}{|\text{n}}_{\text{i}},x_{i}^{R},\text{prior})>\eta^{R}$ at interim, then stop for futility
- If $P(({\text{X}}_{\text{predictive}}^{\text{T}}>x_{c}^{T}-x_{i}^{T}{)}^{\cup }({\text{X}}_{\text{predictive}}^{\text{R}}<x_{c}^{R}-x_{i}^{R})|n_{i},x_{\text{i}}^{\text{T}},x_{i}^{R},\text{prior})>\eta$ at interim, then stop for a combination of poor treatment and/or high toxicity
- If $P(({\text{X}}_{\text{predictive}}^{\text{T}}\leq x_{c}^{T}-x_{i}^{T}{)}^{\cap }({\text{X}}_{\text{predictive}}^{\text{R}}\geq x_{c}^{R}-x_{i}^{R})|n_{i},x_{\text{i}}^{\text{T}},x_{i}^{R},\text{prior})>\zeta )$ at interim, then stop for efficacy
- Recommend further research at final analysis if $P(\Theta^{R}>\theta_{0}^{R}|n,x^{R},\text{prior})>\zeta^{R}$ and $P(\Theta^{T}<\theta_{1}^{T}|n,x^{T},\text{prior})>\zeta^{T}$

where ${\text{X}}_{\text{predictive}}^{\text{T}}$ is the posterior predictive distribution for $n-n_{i}$ patients and $x_{c}^{T}$is the largest $x^{T}$ which satisfies $P(\Theta^{T}<\theta_{1}^{T}|n,x^{T},\text{prior})>\zeta^{T}$ at the end of the trial. Similarly where ${\text{X}}_{\text{predictive}}^{\text{R}}$ is the posterior predictive distribution for $n-n_{i}$ patients and $x_{c}^{R}$ is the smallest $x^{R}$ which satisfies $P(\Theta^{R}>\theta_{0}^{R}|n,x^{R},\text{prior})>\zeta^{R}$ at the end of the trial.

### 6.3 Bayesian decision theory approach

Bayesian decision theory is based on minimising the expected loss of each decision and is a good method for comparing risks and rewards for different strategies and decisions. Berry and Ho^15^ proposed using Bayesian decision theory within a clinical trial setting to make decisions during the trial, building on the work of Raiffa and Schlaifer^16^ and DeGroot.^17^ Berry compared this approach to traditional sequential analysis approaches. As a decision has to be made at interim analysis, it is appropriate to apply Bayesian decision theory to choose between the options.

There are three possible outcomes at each analysis of a trial. Each outcome has costs and benefits. For example, stopping for futility or toxicity is costly if the drug actually works and has an acceptable toxicity profile. These costs and benefits are defined mathematically in a loss function. At the planned analysis, the expected cost for each decision is calculated by computing the expected loss for each decision based on the posterior distribution. The decision with the smallest expected loss is the course of action with the best risk/reward trade-off. Continuing the trial will increase the amount of information, but has intrinsic cost if the drug is toxic or does not work, as well as the monetary cost of running the trial.

Chen and Smith^12^ extended this methodology in a phase II setting with two endpoints. They discussed the use of a two-dimensional region: if the true response-toxicity ($\theta^{R},\theta^{T}$) pair fell within the acceptable region, then further research could be recommended. The three loss functions relate the probability of being within the region (efficacy and no toxicity), the probability of being outside the region (futility and/or toxicity) and the value of collecting additional information traded off against the cost of continuing the trial (continue recruitment). As the region does not have to be square in ($\theta^{R},\theta^{T}$) space, a trade-off can be applied between response and toxicity.^7^ Chen and Smith proposed using an odds ratio for response and toxicity when comparing a treatment with existing treatment options.

When the maximum number of patients is reached, the model can provide a region of uncertainty in which the data still recommend further recruitment. Unlike other methods, the framework does not force a decision to be made on concluding the trial, although it is possible to impose a decision here. In this context, the planned trial is part of a larger trial, censored at the planned maximum number of patients. In a larger trial context, many more patients are needed to make a conclusive decision when the ($\theta^{R},\theta^{T}$) parameters are borderline between regions. Under this methodology, the trial can continue if the observed data places the trial in this region of uncertainty and there is funding available.

This framework has two degrees of freedom. The first balances stopping for efficacy and no toxicity against stopping for futility and/or toxicity (type I vs. type II error). The second balances the cost of collecting information against the cost of stopping incorrectly. The acceptable region also plays a large part in any frequentist properties computed for designs based on this methodology.

**Figure 1.**
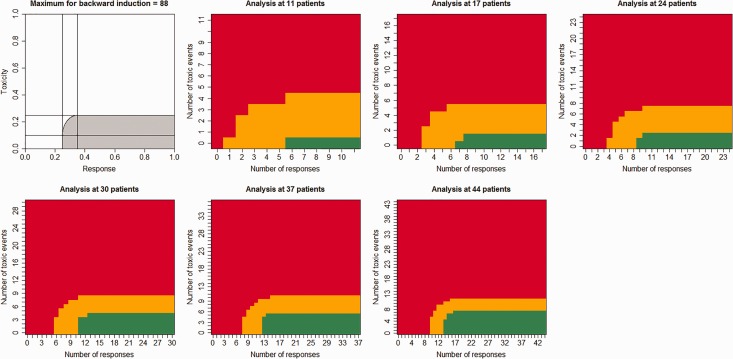
Decision rules for the formal Bayesian decision rule approach. Red: stop the trial for futility. Orange: continue the trial. Green: stop the trial for efficacy.

This method relies on backward induction: the decision at 30 patients relies on the loss for the decision made at 35 patients, whilst the decision made at 35 patients relies on the loss for the decision made at 40 patients. This is due to the continuous treatment loss function, which relies on the critical values used to make the decision at the next interim analysis. Figure 1 shows a proposed acceptable region alongside a resulting design. There are a number of alternatively shaped regions.

## 7 Programmes

The programmes used to perform the analysis for this paper are available for R from the CRAN packages repository in the EurosarcBayes package. The package contains all of the methods discussed in this paper. It also contains the following methods for single endpoint designs: frequentist single stage, Simons two stage,^5^ Lan-deMets alpha spending,^9^ the Bayesian posterior probability approach and the Bayesian posterior likelihood approach. Independent beta priors are implemented for all of the methods.

## 8 Motivating example

LINES is a small phase II trial testing the efficacy and toxicity profile of linsitinib in patients with relapsed and/or refractory Ewing sarcoma (ClinicalTrials.gov registration number: NCT02546544). Linsitinib has been tested in a number of Phase I–III trials,^18–21^ but in very few patients with Ewing sarcoma. Toxic interactions are therefore still a major concern and it is important to stop the trial early for futility or toxicity. As this trial is not geared towards a phase III trial if it is a success, it would also be pertinent to stop early to accelerate more conclusive research.

It is known that Linsitinib (OSI-906) is highly active in embryonic stem cell lines when tested in high throughput screens, but not all cell lines were equally sensitive. For this reason the ability to stop for futility is appropriate.

Two patients per million per year present with Ewing sarcoma.^22^ As the LINES trial is not a first line treatment, the patient pool is smaller, 0.6 per million patients. A multinational study is thus required to get a feasible rate of recruitment. Five countries will have centres, giving a population pool of around 350 million people and a potential patient pool of approximately 210 a year. Each country will have one specialist centre and referrals will be made within that country to the centre. Patients may be referred between countries if there is no participating centre in their home country. It should be feasible to recruit around 30 patients a year. Allowing for opening of centres, the recruiting target was set at 40 patients over 18 months of recruitment. This is a small number of patients, which will constrain possible trial designs.

Within the LINES trial, a response rate of 0.2 ($\theta_{0}^{R}$) is considered unacceptable and a response rate of 0.35 ($\theta_{1}^{R}$) is good. A toxic event rate of 0.1 ($\theta_{1}^{T}$) is considered good, whilst a toxic event rate of 0.3 ($\theta_{0}^{T}$) is unacceptable. These four rates combined to give four scenarios to consider. Operating characteristics can be run for each scenario. When both endpoints are considered, the case ($\theta^{R},\theta^{T}$) = (0.35, 0.1) gives the power, whilst the highest false positive rate for the other three scenarios gives the type I error (alpha). The trial aims to have α=0.1 and power=0.8 (type II error of 0.2) for frequentist designs and equivalently η = 0.9 and ζ = 0.9 for Bayesian designs. The ideal power for a phase II trial is power = 0.9. As there are two endpoints that are assumed to be independent, the overall power is the product of the power for each endpoint.

The data considered in setting target probabilities for response and toxicity included Phase II trials of agents that target components of the same growth regulatory insulin-like growth factor (IGF) pathway.^23–26^ Lisnitinib was expected to improve on these responses because it targets two rather than one receptor (Insulin receptor and IGF1 receptor). Moreover, pre-clinical effectiveness of linsitinib in cell lines suggested at least 50% of cell lines would respond to the agent. There was also considerable data on linsitinib in other cancers,^18–21^ however these results do not easily generalise to Ewing’s sarcoma. For this reason we decided against using informative priors in this case.

To show the full power of the Bayesian methods, we show the effects of both non-informative and informative priors. For this informative prior, we use a weight of 10 patients and set the mean of the beta prior to 0.3 for the response and 0.2 for the toxicity endpoints. This corresponds to a Beta(3, 7) for the response endpoint, and a Beta(2, 8) for the toxicity endpoint.

## 9 Results

Table 1 details the design characteristics for the possible trial designs using non-informative priors. We report the least extreme Bayesian posterior probabilities across interim analyses for each design. The Bayesian posterior probabilities for the frequentist designs are computed in a similar way using the critical values at each analysis and a non-informative Beta(1, 1) prior for both endpoints.

**Table 1.** Comparison of approaches for calculating sample size for the LINES trial using non-informative priors.

Design	Sample size at analysis	Frequentist properties				Bayesian posterior probabilities			
		Type I error	Type II error	Sample size H0	Sample size H1	Futility	Efficacy	Toxicity	Toxicity acceptable
Frequentist single stage	44	0.0895	0.1888	44.00	44.00	0.845	0.948	0.996	0.907
Bryant and Day (optimal)	20,50	0.0965	0.1944	31.01	46.16	0.901	0.929	0.997	0.879
Bryant and Day (minmax)	24,41	0.0977	0.1982	33.19	40.26	0.850	0.938	0.993	0.920
Bayesian posterior probability (single stage)	44	0.1526	0.1161	44.00	44.00	0.910	0.901	0.996	0.907
Bayesian posterior probability (two stage)	22, 44	0.1491	0.1689	35.02	34.95	0.910	0.901	0.977	0.907
Bayesian posterior probability (four stage)	11, 22, 33, 44	0.1261	0.2795	26.02	28.14	0.910	0.901	0.952	0.907
Bayesian posterior probability (six stage)	10, 17, 24, 30, 37, 44	0.1364	0.2680	24.62	26.46	0.910	0.901	0.967	0.907
Bayesian posterior predictive probability (two stage)	22, 44	0.1477	0.1286	36.11	40.60	0.910	0.901	0.996	0.907
Bayesian posterior predictive probability (four stage)	11, 22, 33, 44	0.1426	0.1538	30.92	34.71	0.910	0.901	0.996	0.907
Bayesian posterior predictive probability (six stage)	11, 17, 24, 30, 37, 44	0.1455	0.1606	29.53	32.00	0.910	0.901	0.996	0.907
Bayesian posterior predictive probability (continuous evaluation)	11 to 44, continuous	0.1471	0.1959	24.34	25.12	0.910	0.901	0.990	0.907
Bayesian decision theory (four stage, complete)	11, 22, 33, 44	0.1090	0.2657	24.14	29.16	0.910	0.901	0.996	0.907
Bayesian decision theory (six stage, complete)	11, 17, 24, 30, 37, 44	0.1149	0.2617	24.08	28.20	0.910	0.901	0.996	0.907
Bayesian decision theory (four stage, censored^a^)	11, 22, 33, 44	0.2585^b^	0.3700^b^	27.88	32.30	0.978	0.973	0.996	0.979
Bayesian decision theory (four stage with censored portion)	11, 22, 33, 44, 55, 66, 77, 88	0.0804	0.1741	32.96	37.22	0.958	0.958	0.996	0.927
Bayesian decision theory (six stage, censored^a^)	11, 17, 24, 30, 37, 44	0.2445^c^	0.3633^c^	26.07	30.34	0.976	0.975	0.999	0.976
Bayesian decision theory (six stage with censored portion)	11, 17, 24, 30, 37, 44, 55, 66, 77, 88	0.0819	0.1896	30.75	34.70	0.958	0.958	0.999	0.927

a Trial with 88 patients, censored after 44 patients, with censored interim analysis at 55, 66, 77 and 88 patients. The larger trial is directly below the censored trial.

b The type I error includes inconclusive trials with probability 0.2075 and the type II error includes inconclusive trials with probability 0.2276.

c The type I error includes inconclusive trials with probability 0.1895 and the type II error includes inconclusive trials with probability 0.2006.

We need to check whether each frequentist design meets the Bayesian design constraints (posterior probabilities) and whether each Bayesian design meets the frequentist design constraints (alpha and power).

As the single-stage study design does not have any built-in interim analyses, a trial running this design would expect to recruit the maximum number of patients. Forty-four patients are required to achieve α=0.1 andpower=0.8. Most of the power is spent on the response endpoint, as it is more expensive than the toxicity endpoint. The standard power value for single-endpoint phase II trials is 0.9, which gives a sample size of 61 patients. The Bayesian futility endpoint, which is the posterior probability that the response is less than a certain threshold, is not satisfied for this design, as the calculated probability is 0.845 and the threshold is 0.9 (Table 1).

Bryant and Day’s^6^ design allows stopping early for toxicity and futility. The maximum sample sizes for this design are 50 and 41 in the optimal and minimax designs, respectively. The expected sample sizes are at most 31.0 and 33.2 under one of the three null hypotheses. This approach does not incorporate stopping early for efficacy, which prevents issues when the treatment is showing efficacy whilst there is uncertainty about the rate of toxicity. The Bayesian futility endpoint is also violated for the minmax design, as the calculated futility probability is 0.850 and the required threshold is 0.9 (Table 1).

The prior distribution used for the Bayesian designs is a uniform uninformative prior for both response and toxicity. This prior was also used to compute the Bayesian properties of the frequentist designs. As the prior has the weight of two patients, the Bayesian approach does not significantly gain information over the frequentist design. As there is also a cost to stopping at interim analyses (spending α and power), many of these designs have a higher type I and type II error.

The Bayesian single-stage design contains the same number of patients as the frequentist design. The toxicity endpoint is much easier to satisfy in the Bayesian paradigm than the response endpoint. As a single-endpoint trial, the toxicity endpoint only requires 15 patients, whereas the response endpoint requires 44 patients. The single-stage Bayesian design and the single-stage frequentist design require the same sample size. However they require a different number of responders to report success. As a consequence of this, the frequentist design does not satisfy the Bayesian design constraints (posterior probabilities), and the Bayesian design does not satisfy the frequentist design constraints (alpha and power). The power is shared between the endpoints in the frequentist design, giving the response endpoint a larger share because it is more costly to achieve. In contrast, each endpoint is checked independently against $\eta^{R}$ and $\zeta^{R}$for response and $\eta^{T}$and $\zeta^{T}$ for toxicity in the Bayesian design. The frequentist and Bayesian specifications for the problem differ from one another. This is true for all the designs presented.

The posterior probability and posterior predictive probability designs show that increasing the number of interim analyses increases the type II error and reduces the expected sample size under the frequentist hypotheses.

The posterior predictive probability approach improves on the posterior probability approach by significantly reducing the type I error at the expense of a slightly inflated type II error and similar expected sample sizes.

There are two proposed implementations of the Bayesian decision theory method.^12^ The first is a closed trial design with all decisions made at 44 patients. The second treats the trial as part of a larger trial and censors the second half of the trial. Interim analyses are conducted after 55, 66, 77 and 88 patients have been recruited. There is a region of uncertainty between recommending further research and stopping research at 44 patients. Table 1 shows both versions of the trial and includes the uncertainty in the type I and II errors with a note on the probability of recommending continuing the trial.

Figure 1 shows the decisions that should be made for the censored six-stage Bayesian decision theory trial. The trial only continues if the ($\theta^{R},\theta^{T}$) pair is within the orange regions of the graph. The trial will stop for futility or toxicity if the ($\theta^{R},\theta^{T}$) pair is within the red region and will stop for efficacy if within the green region. Graphs like Figure 1 can be produced for all of the designs mentioned here. For the posterior probability designs, the regions are square with no trade-off between response and toxicity.

The complete trial version has similar properties to the Bayesian posterior probability approach. The censored version censors about 20% of the trial under the frequentist null and alternative hypotheses. The uncensored trial has much smaller type I errors than the other trials. The uncensored trials need very few expected patients, recruiting 5.12 and 4.68 for the four- and six-stage trials, respectively.

Table 2 presents the same methods using the informative prior. The results follow generally the same pattern as those generated with the non-informative prior. The information is borrowed by replacing eight patients as the weight of this prior (ten) is greater than the weight of the non-informative prior (two). The price for adding this information is a small increase in type I and type II errors across all of the methods. If we instead simulated the prior data as a frequentist, we would simulate 46 patients. The type I and type II errors would then closely match the results from the non-informative case across all of the methods.

**Table 2.** Comparison of approaches for calculating sample size for the LINES trial using informative priors. We use a Beta(3,7) prior for response and a Beta(2,8) prior for toxicity.

Design	Sample size at analysis	Frequentist properties				Bayesian posterior probabilities			
		Type I error	Type II error	Sample size H0	Sample size H1	Futility	Efficacy	Toxicity	Toxicity acceptable
Bayesian posterior probability (single stage)	36	0.1755	0.1451	36	36	0.91	0.901	0.996	0.907
Bayesian posterior probability (two stage)	18,36	0.1559	0.2257	29.15	27.89	0.91	0.901	0.953	0.907
Bayesian posterior probability (four stage)	9,18,27,36	0.1497	0.2567	24.29	24.29	0.91	0.901	0.953	0.907
Bayesian posterior probability (six stage)	9,15,20,25,30,36	0.1508	0.2659	21.32	22.71	0.91	0.901	0.952	0.907
Bayesian posterior likelihood (two stage)	18,36	0.1662	0.1539	30.81	33.25	0.91	0.901	0.996	0.907
Bayesian posterior likelihood (four stage)	9,15,20,25,30,36	0.1595	0.1684	25.79	27.02	0.91	0.901	0.996	0.907
Bayesian posterior likelihood (six stage)	9,15,20,25,30,36	0.1595	0.1684	25.79	27.02	0.91	0.901	0.996	0.907
Bayesian posterior likelihood (continuous evaluation)	28 analyses between 9 and 36	0.1604	0.1921	22.1	22.56	0.91	0.901	0.99	0.907
Bayesian loss function (four stage, complete)	9,18,27,36	0.1301	0.5727	17.57	25.64	0.922	0.948	0.972	0.992
Bayesian loss function (six stage, complete)	9,15,20,25,30,36	0.0747	0.4695	16.17	23.54	0.91	0.901	0.972	0.953
Bayesian loss function (four stage, censored^a^)	9,18,27,36	0.2149^b^	0.6203^b^	20.26	28.07	0.967	0.975	0.994	0.997
Bayesian loss function (four stage with censored portion)	9,18,27,36,45, 54,63,72	0.0564	0.3192	24.25	34.44	0.947	0.956	0.994	0.954
Bayesian loss function (six stage, censored^a^)	9,15,20,25,30,36	0.2066^c^	0.6201^c^	19.36	27.16	0.97	0.975	0.993	0.997
Bayesian loss function (six stage with censored portion)	9,15,20,25,30, 36,45,54,63,72	0.0559	0.341	23.18	33.09	0.947	0.956	0.993	0.954

a Trial with 72 patients, censored after 36 patients, with censored interim analysis at 45, 54, 63 and 72 patients. The larger trial is directly below the censored trial.

b The type I error includes inconclusive trials with probability 0.2075 and the type II error includes inconclusive trials with probability 0.2026.

c The type I error includes inconclusive trials with probability 0.1823 and the type II error includes inconclusive trials with probability 0.3209.

The final design for the LINES trial was a compromise between the methodology discussed here and the design originally approved by Ethics. The final design was based on the posterior probability approach with seven interim analyses, but was limited to 40 total patients. Without other constraints, we would have recommended the Bayesian posterior predictive probability approach with six interim analyses.

## 10 Limitations

Considering the frequentist and Bayesian properties increases the number of properties and the number of optimisation approaches for a given design increases. All of the designs had a natural trade-off in frequentist properties between type I error, type II error and expected sample size under the null and alternative hypotheses.

In the Bayesian paradigm, the success of the trial could have been defined around similar regions used in the Bayesian decision theory approach. The proposed approach for the Bayesian posterior and posterior predictive approaches can be compared to square regions that require the posterior probability to be greater than 0.81. This is a simplification: if a region is used, a trade-off between endpoints is allowed, as in the frequentist designs. This would more closely match the frequentist design.

This paper does not discuss the use of other non-informative prior distributions (such as the improper prior Beta(0,0) or Jeffrey’s prior ($\text{Beta}(\frac{1}{2},\frac{1}{2})$). It can be argued that the Beta(1,1) prior is informative, as it suggests that one success and one failure are observed before commencing the trial. The improper Beta(0,0) prior is closer to a zero information prior, which is more appropriate from a frequentist perspective. The Beta(1,1) prior is also very optimistic for the response endpoint and very pessimistic for the toxicity endpoint.

## 11 Conclusions

In this motivating example, a non-informative prior was used. However, should more information be available in other cases, then the performance of the informative prior should be compared against that of the non-informative prior, as done here. An efficiency gain may be possible in these cases.

No single design approach was optimal under all of the studied conditions. All of the considered designs showed strengths and weaknesses (Table 3). Any given trial will have a number of possible designs that should be considered and compared.

**Table 3.** Summary of conclusions.

	Methodology	Advantages	Disadvantages
	Frequentist	Formal control of type I and type II error	Increased sample size
	Bayesian	Inclusion of informative prior information. Particularly useful when a previous trial has data that can be used to inform the prior Smaller sample size Continual assessment is possible	Informative priors reduce sample size, which will increase the trial type I and type II error
Design			
	Single stage	Trial is simple to design, run and analyse	
	Multistage – stop early for futility	If treatment is futile, the expected number of patients exposed is smaller	Small changes in design affect the trial properties
	Multistage – stop early for efficacy	If treatment if efficacious, the expected number of patients required is smaller accelerating research	Small changes in design affect the trial properties
Single-stage	Frequentist	Easy to run and analyse	
Bryant and Day two-stage design	Frequentist	Inclusion of a second endpoint over Simon’s two-stage design	Type I and type II errors are higher for the same sample size
Posterior probability	Bayesian	Smallest expected sample sizes	Highest type I and type II errors tend to be a non-significant chance of stopping prematurely at the first few interim analysis
Posterior predictive probability	Bayesian	Good balance between expected sample size and certainty of results	
Bayes Decision theory	Bayesian	Easy to extend a trial if the results are uncertain	Complicated to design

Adding an additional endpoint either requires more patients or has a negative impact on the design properties, regardless of the underlying methodology. Having the extra endpoint causes more error possibilities.

The Bayesian approaches do not provide value-added changes to frequentist designs unless informative prior information is incorporated. The Bayesian methods provide significant flexibility in trial design by allowing both multiple interim analyses and multiple endpoints. In contrast, the frequentist methods are capable of multiple interim analyses or multiple endpoints, but currently no work has been done to accommodate both at the same time. Trials can be designed in a frequentist or Bayesian setting and compared using both frequentist and Bayesian properties. This ability to directly compare different designs is particularly important from a regulatory point of view, as fair decisions about a drug’s suitability are required regardless of the trial design or methodology used.

The posterior predictive probability approach is the most balanced Bayesian approach for the LINES trial considered here. It has a good trade-off between confidence in the results when stopping early and the expected number of patients needed to recruit. In contrast, the posterior probability approach tends to recommend stopping too early. The Bayesian decision theoretic approach would be a suitable design if it were possible to request further funding and additional patients could be recruited in sensible time frames in the event of borderline results. In the example discussed, this would occur 20–40% of the time (if between the null and alternative hypotheses) and would only increase the expected sample size by 4–5 patients under the frequentist null and alternative hypotheses.

It is possible to design a trial in either a frequentist or Bayesian setting, then plan to analyse it in the other setting. Any given trial design can define null and alternative hypotheses in the frequentist setting and can define Bayesian posterior probabilities. Thus, frequentists can use Bayesian designs, and vice versa. Regardless of the design approach, the trial will be a series of analyses with critical regions for each analysis.

Without prior information, Bayesian designs do not immediately add value over equivalent frequentist designs. Deciding between these methodologies should be done in light of key prior information or the intention for further research. If a future trial following successful results intends to use the prior information from the first trial, then maintaining a Bayesian approach throughout allows the data from the first trial to be used. These results would be discarded in the frequentist setting, unless the trial was formally designed as a phase I/II or phase II/III trial. The two design approaches will suggest similar sample sizes for the first trial. However, under the frequentist approach, the patients in the first trial will be discarded and will have to be replaced with new patients in the second trial. Under the Bayesian approach, the patients in the first trial will not be discarded, resulting in a huge saving in required sample size for future trials.
